# Quantification of luminol chemiluminescence: a tool for estimating the postmortem interval of skeletal human remains

**DOI:** 10.1007/s00414-025-03713-w

**Published:** 2026-01-19

**Authors:** Catarina Ermida, Miguel Morgado, Eugénia Cunha, Maria Teresa Ferreira

**Affiliations:** 1 Centre for Functional Ecology, Laboratory of Forensic Anthropology, Department of Life Sciences, University of Coimbra, Calçada Martim de Freitas, Coimbra, 3000-456 Portugal; 2 LIBPhys-UC, Department of Physics, Faculty of Sciences and Technology, University of Coimbra, Rua Larga, Coimbra, 3004-516 Portugal; 3 CIBIT - Coimbra Institute for Biomedical Imaging and Translational Research, Institute for Nuclear Sciences Applied to Health (ICNAS), University of Coimbra, Coimbra, 3000-548 Portugal; 4 National Institute of Legal Medicine and Forensic Sciences (INMLCF), South Branch, Rua Manuel Bento de Sousa 3, Lisboa, 1150-334 Portugal

**Keywords:** Human skeletal remains, Luminol quantification, Chemiluminescence, Time since death, Forensic anthropology

## Abstract

Postmortem interval (PMI) estimation is mandatory in any forensic investigation. Although it remains a very challenging task, particularly in skeletonized human remains. Luminol chemiluminescence has been explored as a tool for PMI estimation, however, its application requires further experimental validation to ensure accuracy and reproducibility. The present research aims to increase the objectivity of PMI estimation by quantifying the light emission from the luminol chemiluminescence reaction. For this purpose, a sample of clavicles collected from 24 adult individuals of both sexes, with known PMI ranging from 7 to approximately 500 years, was selected. After being reduced to powder, the samples were analyzed on a CCD camera with an image intensifier, and the reaction Ipeak was measured. The results obtained revealed a clear inverse relationship between PMI and Ipeak values. A segmented regression analysis identified a statistically supported threshold at approximately 30 years postmortem, distinguishing a variable early postmortem phase from a long-term phase characterized by consistently low and minimally variable chemiluminescence intensities. This pilot study supports the validity of quantifying the luminol chemiluminescence reaction as a feasible and informative presumptive screening tool for PMI estimation, with potential applicability in distinguishing remains of forensic interest from those of archaeological relevance.

## Introduction

Estimating the postmortem interval (PMI) constitutes a critical challenge in forensic sciences, as it determines the time elapsed since death and helps establish the forensic relevance of human remains [1]. This parameter is especially significant when dealing with human skeletonized remains recovered under suspicious circumstances, as it distinguishes forensic cases from archaeological findings, which have distinct legal implications [2–4]. The relevance of an accurate PMI for identification is sometimes underestimated. Primarily, during the confrontation phase with the missing persons’ list, it’s crucial to have a precise estimate. In other words, more identifications can be reached if the methods for PMI are improved. However, establishing an accurate PMI estimation remains a complex task, due to the variability in the decomposition process, influenced by environmental, anthropogenic, and intrinsic factors, such as temperature, soil pH, humidity, and burial context [5–7]. The challenge is further increased by practical constraints, including cost, time limitations, and the state of preservation of the remains [8]. Consequently, forensic anthropologists require cost-effective and reliable methods for PMI estimation that align with scientific standards, such as the Daubert criteria [8, 9].

Among the existing methods, radiocarbon dating is one of the few reliable techniques for estimating PMI in skeletonized remains, yet it is expensive and technically demanding [10]. Recently, the use of luminol chemiluminescence has gained attention as a simple and affordable alternative for distinguishing forensic from archaeological remains [11–13]. Traditionally used for detecting blood traces in forensic investigations, luminol reacts with the iron ions in hemoglobin, emitting a blue chemiluminescent light when exposed to an oxidizing agent such as hydrogen peroxide [14–16]. While highly sensitive, luminol is susceptible to false positives caused by substances such as bleach, plant peroxidases, and some metals [17–19].

Studies have expanded the application of luminol to PMI estimation by correlating its reaction intensity with the persistence of hemoglobin in bone tissue [6, 12, 13, 20]. Hemoglobin can be preserved within bone structure for extended periods due to its rigidity and mineral composition, but it degrades over time, leading to a decrease in chemiluminescence intensity [12, 21]. Consequently, a positive luminol chemiluminescence reaction is more common in recent remains. In contrast, older bones tend to exhibit weaker or no chemiluminescence [13, 20], suggesting that luminol testing can serve as a preliminary indicator of forensic relevance, prompting more sophisticated analyses when necessary.

Despite its potential, the luminol method requires additional validation to ensure accuracy and reproducibility across diverse forensic scenarios. Studies have demonstrated its effectiveness across different bone types and preservation conditions, though some findings suggest that non-intact bones may yield variable results [6, 17]. Additionally, Sarabia et al. (2018) proposed a quantitative approach using a luminometer to measure chemiluminescence intensity in relative light units (RLU), enabling more precise PMI estimation. Although previous studies using visual scales have already shown excellent inter and intraobserver agreement [11], reinforcing the method’s reproducibility even before the adoption of quantitative techniques, this quantitative method may further enhance the reliability of PMI estimation, bringing it closer to forensic applicability under standardized criteria.

Given these considerations, this study aims to refine previous research by expanding the knowledge about this technique to assess an accurate PMI estimation. By adopting a quantitative approach, this research seeks to enhance objectivity in PMI estimation through luminol chemiluminescence. This methodological refinement plans to improve reproducibility, minimize observer bias, and ensure alignment with forensic standards such as the Daubert criteria.

## Materials and methods

**Fig. 1:**
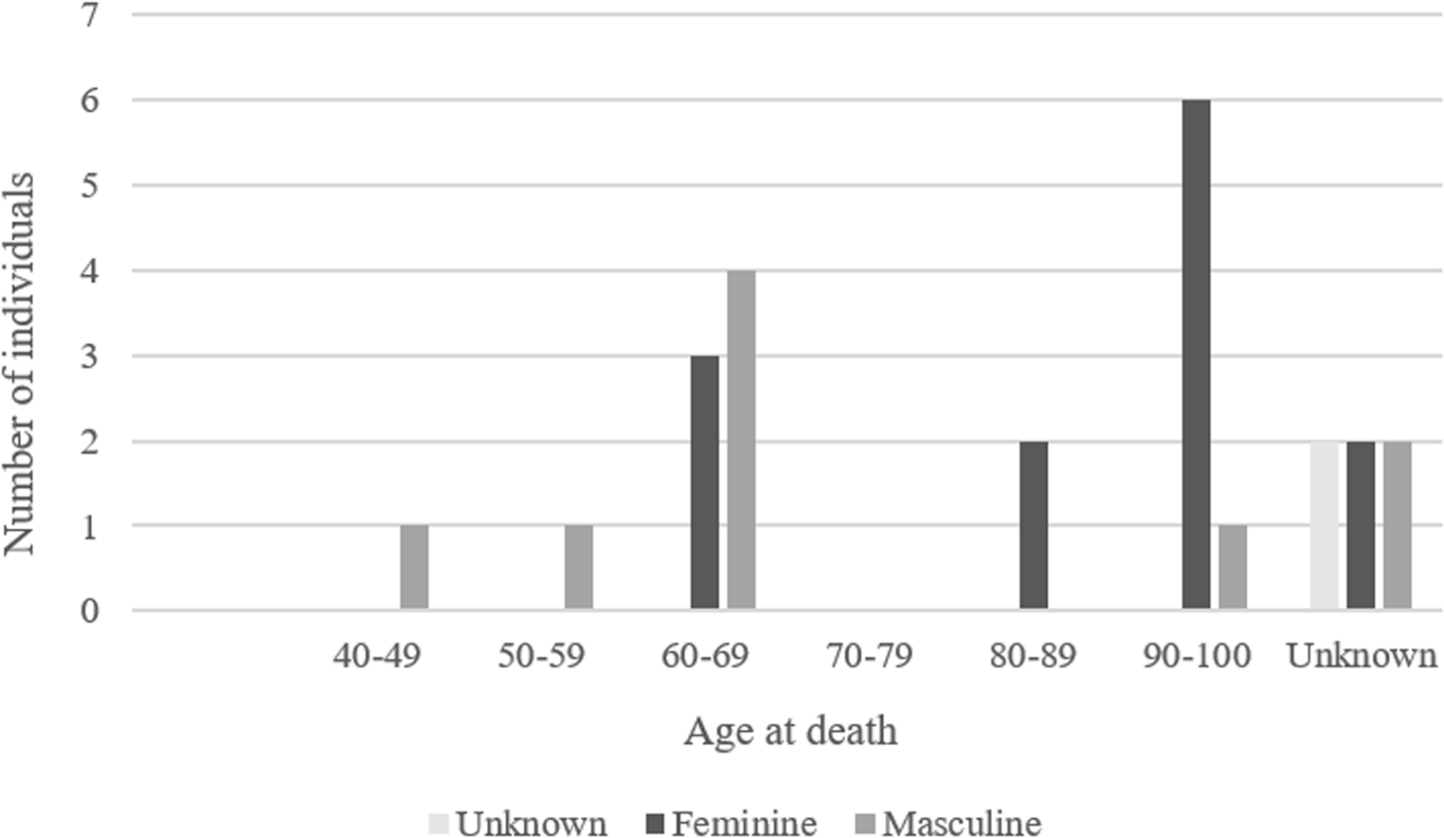
Sample distribution according to sex and age

Our study involved a sample of 24 human clavicles from adult individuals of both sexes, with ages at death ranging from 37 to 99 years (mean: 75.8, median: 74.5) (Fig. 1). Bones that had no ante or perimortem trauma or identifiable pathology were selected to ensure consistency in the sample. The clavicles were sourced from various origins: a fresh bone from an autopsy performed at the Portuguese National Institute of Legal Medicine and Forensic Sciences (INMLCF.IP.), Centre Branch (approved by the Ethics Commission of the INMLCF.IP.) (*n* = 1); dry skeletal remains from the 21 st Century Identified Skeletal Collection (approved by the Ethics Committee of the Faculty of Medicine of the University of Coimbra, CE_026.2016) [22] (*n* = 15); dry skeletal remains from autopsies performed at the INMLCF.IP., South Branch, from the 20th century (approved by the Ethics Commission of the INMLCF.IP., CE-21/2021) (*n* = 7); and one dry clavicle from the Valle da Gafaria osteoarchaeological collection [23] (*n* = 1). The postmortem interval of these samples ranged from 7 to approximately 500 years, when the technique was applied (Table 1).

**Table 1:** Sample distribution according to age, sex and PMI

Sample	Age	Sex	PMI (years)
1	64	F	7.40
2	96	M	10.24
3	80	F	11.00
4	98	F	12.48
5	55	M	13.28
6	92	F	14.05
7	69	M	15.22
8	60	M	16.00
9	99	F	17.00
10	61	F	19.70
11	97	F	20.00
12	60	M	21.66
13	90	F	22.70
14	90	F	23.20
15	85	F	24.35
16	43	M	26.26
17	Unknown	Unknown	27.00
18	Unknown	M	34.00
19	Unknown	F	38.00
20	60	F	40.00
21	Unknown	M	42.00
22	Unknown	Unknown	46.00
23	65	M	53.00
24	Unknown	F	~ 500.00

The clavicle obtained from the INMLCF.IP., after checking RENNDA – National Register of Non-Donors – and receiving approval from the Ethics Commission, was previously processed, reduced to powder, and used in an earlier study [21]. It was stored at 4 °C in a labeled zip-lock bag until the study conditions were met, then defrosted, water macerated, and manually cleaned using sandpaper and brushes. A small portion was cut with a power saw, pulverized in a liquid nitrogen mill, and collected in an Eppendorf tube (50 mg).

The remaining clavicles (*n* = 23) were cleaned with brushes and sandpaper. The diagenetic influence was minimized by focusing on cortical bone, ensuring thorough and precise sample preparation. A scalpel was used to reduce the bone to powder, and 50 mg from each clavicle was stored in labeled Eppendorf tubes.

Luminol 16oz solution was prepared according to the label instructions and used within 3 h after preparation. The same operator conducted the entire process of sample preparation and luminol application. To minimize the potential irritant effects of luminol, the operator wore protective gear, including gloves and protective clothing, during the spraying process.

The analyses were performed using a Orca-285 CCD camera (Hamamatsu Photonics K.K., Shizuoka, Japan) with a C9546 image intensifier. Each clavicle powder sample (50 mg) was placed in the center of a petri dish, which was positioned aligned with the camera axis at its focal plane. The luminol was then applied in spray at room temperature in a completely dark room, and the intensity of the chemiluminescence reaction, measured in arbitrary units (A.U.), was recorded for 15 min, with partial measurements taken every 15 s, starting with the first measurement at 15 s, as this was the time reported by Sarabia and team (2018) for a positive response. As for more recent samples, a more intense reaction was expected, while older samples were supposed to exhibit a weaker reaction, the exposure time was adjusted for each sample PMI to allow the capture of the reaction’s light emission with the maximum possible precision without losing linearity (please refer to Table 2 for details). Subsequently, a correction factor was applied to ensure the consistency of the results. The reaction intensity Ipeak (Table 2) corresponds to the maximum emission of the sample. The Ipeak calculation was done using MATLAB version R2024b (The MathWorks, Inc., Natick, Massachusetts).

**Table 2:** Light emission measurements of the luminol chemiluminescence reaction according to PMI and correction factors according to exposure time and PMI (in years)

Sample	PMI (years)	Ipeak	Exposure time (s)	Correction factor
1	7.40	9142.70	1	5
2	10.24	2207.65	1	5
3	11.00	4437.25	1	5
4	12.48	5617.40	1	5
5	13.28	7928.28	1	5
6	14.05	2506.60	1	5
7	15.22	2123.01	1	5
8	16.00	1359.88	2	2.5
9	17.00	1530.92	2	2.5
10	19.70	1308.67	2	2.5
11	20.00	1053.97	2	2.5
12	21.66	563.04	3	1.67
13	22.70	704.52	3	1.67
14	23.20	369.25	4	1.25
15	24.35	394.93	4	1.25
16	26.26	505.56	4	1.25
17	27.00	608.53	4	1.25
18	34.00	277.93	4	1.25
19	38.00	252.70	5	1
20	40.00	228.36	5	1
21	42.00	240.08	5	1
22	46.00	230.32	5	1
23	53.00	211.20	5	1
24	500.00	218.68	5	1

The results were analyzed and statistics were carried out using Excel (Microsoft, Redmond, Washington), and MATLAB to assess the value of the luminol chemiluminescence technique as a presumptive tool for estimating time since death. The statistical analysis did not consider variations related to sex and age-at-death, aligning with previous literature [6, 13, 20].

## Results

**Fig. 2:**
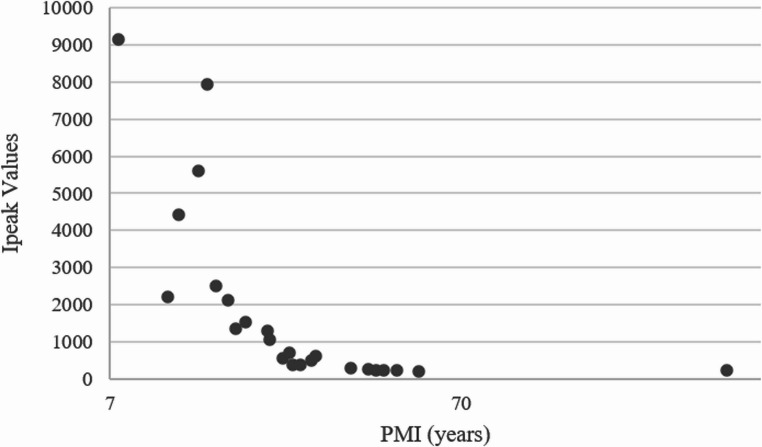
Luminol chemiluminescence quantitative measurements (Ipeak) according to PMI. PMI is represented on a base-10 logarithmic scale

The results of the experiments quantifying the luminol reaction’s light emission, illustrating the relationship between PMI and chemiluminescence intensity (Ipeak), are summarized in Table 2; Fig. 2.

It’s worth noting that the Ipeak values are higher for samples with a lower PMI and decrease as PMI increases. This aligns with the results from existing literature, showing that more recent samples exhibit a more intense reaction, while older samples show a weaker response. However, some samples do not follow a monotonic pattern, with the most notable examples being samples 2, 3, and 4, which exhibit significantly lower Ipeak values compared to sample 1. In these cases, instead of decreasing over time, their Ipeak values show an unexpected increase. Additionally, samples 1 and 5 exhibit a higher Ipeak value when compared to the rest of the dataset. From 14 years PMI onwards, the decline in intensity is not consistent, with some small value oscillations around PMI values of 17, 22, 26 and 27 years. Above a 20-year PMI, all Ipeak values are below 1000 A.U. From a 30-year PMI onward, the IPeak values remained consistently below 300 A.U. Additionally, the sample with a PMI of approximately 500 years has an Ipeak value similar to samples with PMIs between 40 and 53 years, suggesting a possible plateau in residual intensity that warrants further investigation. For statistical purposes, the 500-year PMI sample was not included in the analysis. The large temporal gap between this point (500 years) and the preceding sample (53 years) would require extrapolating across an interval with no intermediate data, which would artificially inflate the model’s uncertainty in this range.

**Fig. 3:**
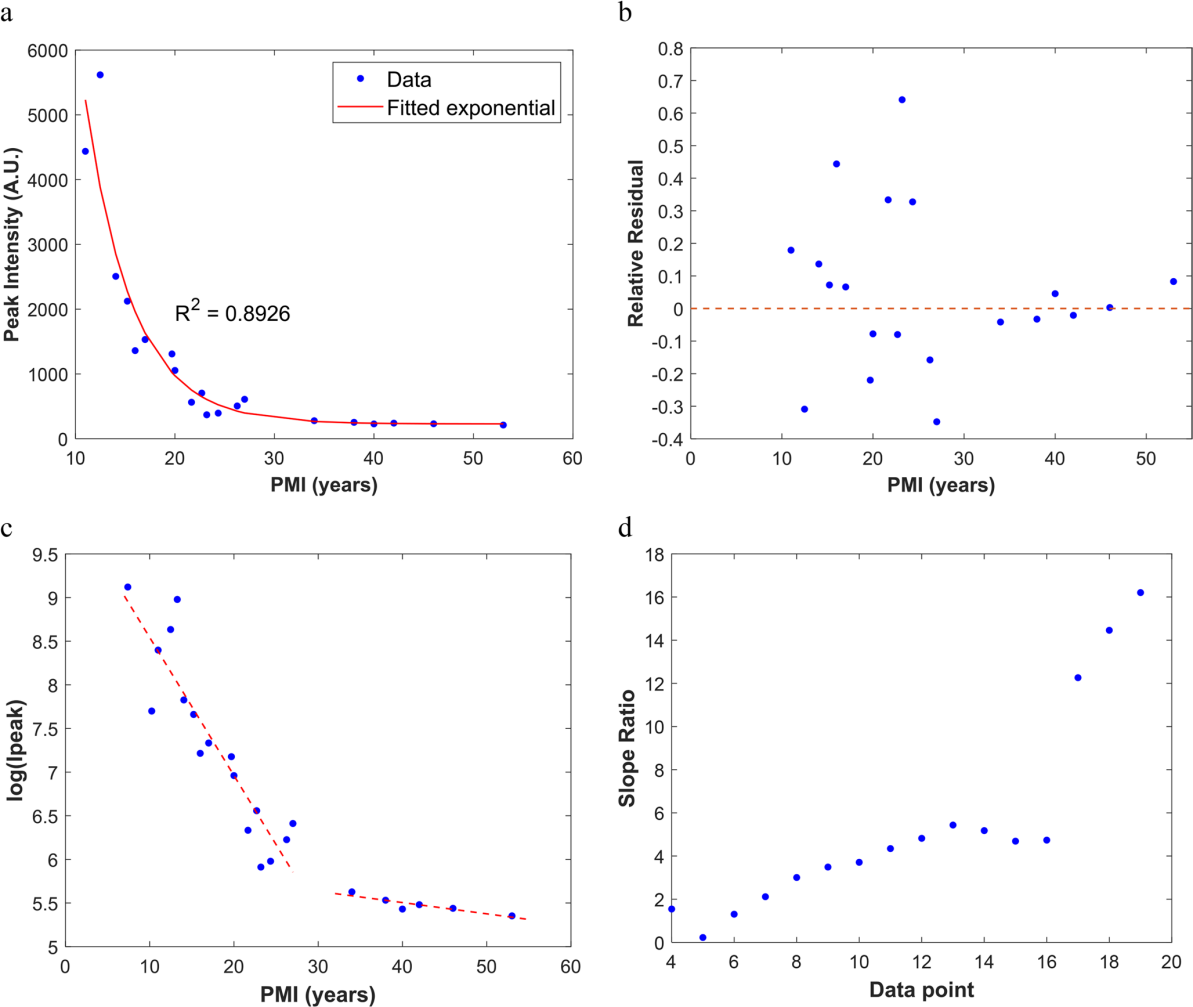
Exploratory and diagnostic analyses supporting the presence of two distinct behavioural phases in the relationship between Peak Intensity and PMI. (**a**) Initial exponential fit after removal of outliers (Cook’s distance), showing the overall decreasing trend. (**b**) Residual analysis, with large residuals for PMIs < 30 years and minimal variation for higher PMIs. (**c**) Log-transformed plot highlighting two visually distinct regions. (**d**) Changepoint analysis indicates an optimal transition between 27 and 34 years (data points 17 and 18)

To explore the quantitative relationship between peak intensity and the postmortem interval (Table 2) an initial exponential decay regression model was fitted to the dataset. Before performing this adjustment, an outlier analysis based on Cook’s distance was conducted, and three points (samples 1, 2 and 5) were excluded from this first exploratory fit. The resulting exponential model (Fig. 3a) showed a coefficient of determination (R²) of 0.8926. Although this preliminary fit visually captured the overall decreasing trend, a residual analysis (Fig. 3b) revealed clear systematic bias. The residuals for PMIs < 30 years were large and non-random, while those for PMIs > 30 years were small and clustered close to zero. This behavior indicates that a single model is inadequate, as it cannot satisfactorily describe the entire PMI range, and suggests a structural changepoint with two distinct behavioral phases. Given the residuals pattern, the hypothesis that two segments better described the relationship between chemiluminescence intensity and PMI was tested. A log-transformed exploratory plot (Fig. 3c) supported this interpretation, visually revealing two distinct regions: an initial phase characterized by a steep decrease in Ipeak for PMIs < 30 years, followed by a phase of lower variation for higher PMIs. To formally evaluate this hypothesis, a segmented regression with a grid search to identify the optimal breakpoint. was performed. Briefly, after the logarithmic transformation, the data was split in two segments and iterated the changepoint between data points 5 and 19. For each iteration, a straight line was fitted to each segment and the ratio of their slopes was plotted. (Fig. 3d). The optimal breakpoint was identified by the highest variation in the slope ratio. This breakpoint is biologically plausible and aligns with the visual inspection of the data.

Following the exploratory and diagnostic evidence presented above, the data analysis procedure returned to the original dataset (*n* = 23), excluding only the 500-year PMI sample for the reasons previously described. A new outlier assessment was performed using Cook’s distance, this time applied separately to each of the two segments. Three points were identified and removed in the first segment, while no influential observations were detected in the second segment. A segmented regression model was fitted to the dataset, using the breakpoint identified in the changepoint analysis. The first segment (PMI < 30 years) was adjusted using an exponential decay function:

$$\:y={A}_{1}*\mathrm{e}\mathrm{x}\mathrm{p}(\:-\frac{x-{x}_{0}}{{t}_{1}})$$

To avoid overfitting, the second segment (PMI > 30 years) was fitted using a linear function:

$$\:y={A}_{2}+{B}_{2}x$$

**Fig. 4:**
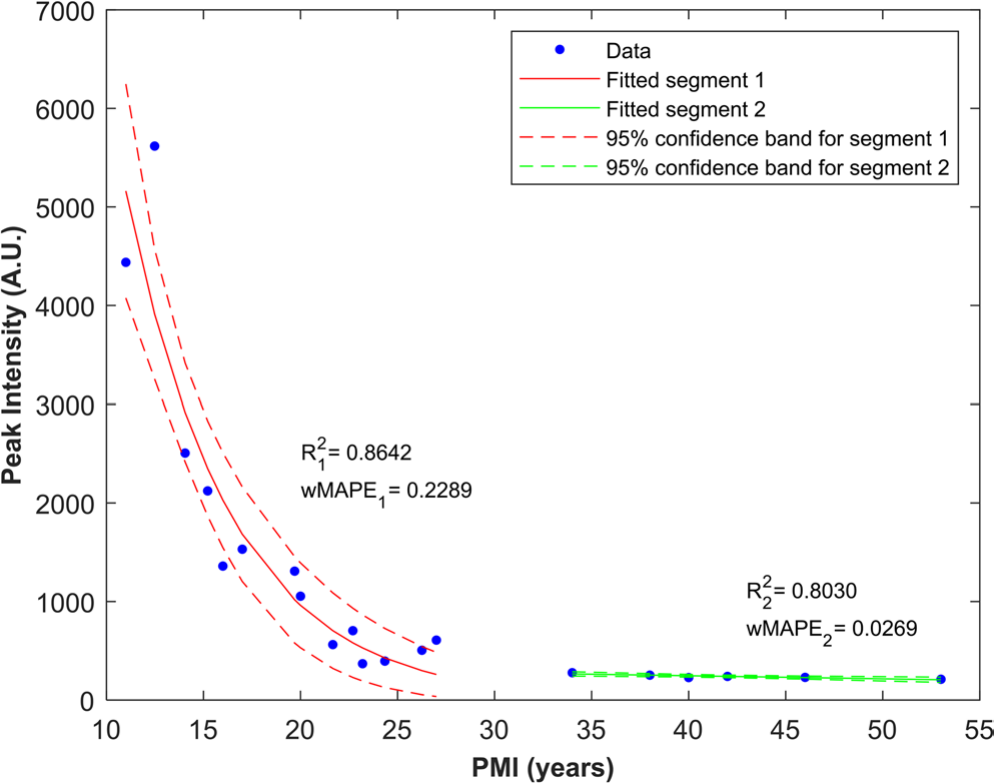
Segmented regression model of luminol chemiluminescence peak intensity with increasing PMI. The first segment (PMI < 30 years) follows an exponential decay function, while the second segment (PMI > 30 years) is represented by a linear model. Dashed areas represent 95% confidence bands

The resulting model is shown in Fig. 4.

In Fig. 4, a marked decline in Ipeak intensity is observed as PMI increases, consistent with the expected hemoglobin degradation trend over time. In the first segment, the exponential model achieved a coefficient of determination (R²) of 0.8642, and a weighted Mean Absolute Percentage Error (wMAPE) of 0.2289. The linear segment yielded an R² of 0.8030, and a wMAPE of 0.0269. The exponential segment shows a steep decrease in Ipeak across the early PMI range, with wide confidence bands reflecting the higher variability observed in this interval. In contrast, the linear segment describes the very low-variation behavior observed for PMIs > 30 years, with narrow confidence intervals. Overall, the first segment (PMI < 30 years) demonstrates a strong inverse relationship between the time since death and the luminol chemiluminescence intensity, while the second segment (PMI > 30 years) reflects a phase of minimal variation over time. Together, the two segments capture the transition from a highly variable early degradation phase to a more uniform long-term pattern, suggested by the exploratory plots, residual structure, and changepoint analysis.

Fitting parameters and corresponding confidence intervals for both segments of the regression model are presented in Table 3.

**Table 3:** Fitting parameters and corresponding confidence intervals for the segmented regression model

Segment	Parameter	Estimate	CI (Lower-Upper)
Segment 1 (PMI < 30 years)	A_1_	8377,260	8377,259–8377,261
	t_1_	5,36	3,58 − 7,13
	x_0_	8,41	6,60 − 10,21
Segment 2 (PMI > 30 years)	A_2_	−3,11	−5,25 - −0,97
	B_2_	371,2	280,1–462,3

To evaluate the predictive performance of the segmented regression model, a Leave-One-Out Cross-Validation (LOOCV) procedure was applied separately to each segment of the model. The predictive error was quantified using the Root Mean Square Percentage Error (RMSPE). The higher-PMI segment showed high predictive accuracy, with an RMSPE of 6,16%, confirming the stability and robustness of the linear model in the long-term region of the curve. In contrast, the lower-PMI segment yielded a higher RMSPE of 37,32%, reflecting the natural biological and taphonomic variability of this early phase. Overall, the LOOCV results demonstrate that the segmented model appropriately isolates a highly stable and predictable long-term phase from a complex and inherently variable early phase. The strong predictive performance in the PMI > 30 region confirms that the low-variation long-term behavior is statistically robust, while highlighting the inherent limitations in predicting Ipeak values at low PMIs.

## Discussion

Dating the death of skeletonized human remains has been one of the significant challenges in forensic anthropology, as bones are subjected to a wide range of environmental and individual factors that influence their decomposition and diagenesis. As a result, estimating time since death with accuracy is particularly difficult, even when the objective is simply to differentiate between recent remains and archaeological ones. Although several methods are currently available for PMI estimation, many of them lack precision, are technically complex, costly, and not widely accessible [20, 24]. In this context, the present study aimed to explore a complementary quantitative approach for PMI estimation that is both low-cost and simple to apply, yet effective.

This work represents a pilot study designed to evaluate the feasibility of quantitatively measuring luminol chemiluminescence in human bone to estimate the postmortem interval. As such, it involved a limited but as representative as possible sample, providing preliminary insights into the potential and constraints of this quantitative approach while establishing a foundation for future, larger-scale research.

The results obtained in this study demonstrate a clear decrease in luminol chemiluminescence intensity (Ipeak) with increasing time since death. This decline, most prominent in the first 30 years after death, reflects the progressive degradation of hemoglobin derived iron compounds in the clavicle sample. For this reason, as the PMI increases, the luminol reaction intensity diminishes, resulting in lower Ipeak values. Overall, Ipeak values decrease markedly in recent PMIs, whereas older samples exhibit much lower and more uniform intensities.

The initial attempt to model the entire dataset using a single exponential decay function (Fig. 3a) revealed significant structural limitations. Although the overall trend showed a decline in peak intensity with increasing PMI, the residual analysis (Fig. 3b) demonstrated clear heteroscedasticity: samples with PMIs below 30 years exhibited large, systematic deviations from the fitted curve, whereas samples with higher PMIs clustered tightly around very low values. This pattern indicated that the dataset did not follow a single continuous exponential trajectory but rather reflected two distinct phases of degradation.

To address these structural limitations, a segmented regression approach was adopted. This model allowed the dataset to be partitioned into two distinct segments, separated by a changepoint supported both by visual inspection (Fig. 3c) and by statistical analysis (Fig. 3d), at approximately 30 years postmortem. Below this threshold, Ipeak values followed a steep exponential decline, whereas above it the data were best described by a low-variation trend. This segmentation provides a more accurate representation of the underlying degradation process than a single continuous model and is consistent with both the residual structure and the exploratory analyses. In fact, the identification of a statistically supported changepoint at approximately 30 years postmortem, which represents a practical threshold in the behaviour of luminol chemiluminescence intensity, constitutes a central outcome of this study. This threshold reflects a shift from a highly variable early postmortem phase to a long-term phase characterized by consistently low and minimally variable Ipeak values. From a forensic perspective, this distinction is particularly relevant, as it aligns with the practical need to differentiate remains that may fall within a forensic timeframe from those more likely to be of archaeological origin. Importantly, this threshold emerges from the segmented modelling approach and is supported by the residuals structure, the changepoint analysis, and the cross-validation.

The exponential behavior observed in the first segment reflects the rapid and irregular degradation of hemoglobin-derived compounds during the early postmortem period. In contrast, the second segment represents a long-term phase characterized by markedly reduced variation and low Ipeak values (below 300 A.U.), suggesting that hemoglobin components have largely degraded at minimal levels. The linear function used to model this second segment was selected for statistical robustness, namely to avoid overfitting, given the small number of available samples (*n* = 6). However, this choice does not rule out the possibility that the second segment follows a non-linear trend; rather, it reflects a statistical decision based on the current dataset. Future studies, including a larger number of older samples, should further investigate the underlying pattern of this segment.

The performance of the segmented model is further supported by the cross-validation results (LOOCV), which indicate a low predictive error in the second segment (6.16%) and confirm that separating the early highly variable phase from the later low-variation phase improves the overall performance of the model. Together, these findings demonstrate that the relationship between luminol chemiluminescence intensity and PMI is best interpreted as a biphasic process and support its value as a presumptive screening tool for distinguishing skeletonized human remains of forensic interest from archeological ones.

Despite the overall consistency of the segmented model, variability remained particularly pronounced among the clavicles with the lowest PMIs. This variability was reflected not only in the residual structure but also in the outlier diagnostics, with three samples with low PMIs (samples 1, 2 and 5) exceeding Cook’s distance thresholds and therefore being excluded. Such heterogeneity in the results obtained from the samples with early PMIs is likely related to the influence of taphonomic factors, including intrinsic factors (such as the individual’s physical condition, age or bone structure) and extrinsic variables (such as burial environment, temperature, humidity and oxygen exposure), which can affect the initial rate and trajectory of hemoglobin degradation. These elements tend to exert a profound influence during the initial stages of decomposition, when molecular components such as hemoglobin are more abundant and susceptible to environmental conditions.

In a previous study conducted by our team [25], the intensity of the luminol chemiluminescence reaction was qualitatively evaluated in a human bone sample buried under different environmental conditions. It was concluded that results obtained through the application of the luminol technique for estimating time since death could be influenced by taphonomic factors such as pH and type of soil, temperature and humidity, reinforcing the importance of considering the context in which a body is found when applying this method. However, in the present study, only one of the three clavicles excluded as statistical outliers had a known and controlled postmortem history, having been collected during an autopsy conducted at the Portuguese National Institute of Legal Medicine and Forensic Sciences and subsequently kept under monitored conditions. In contrast, the remaining two, although also of recent origin, were sourced from dry skeletal remains from the 21 st Century Identified Skeletal Collection, and underwent decomposition under environmental conditions that are not fully documented or controlled (although the characteristics of the graves and the burial ritual are similar, as they originated in the same cemetery). For this reason, the exclusion of these outliers cannot be explained based on the decomposition conditions of this subsample.

In summary, the luminol chemiluminescence results may be influenced by multiple taphonomic variables, such as burial conditions, climate, and postmortem handling, which can affect the survival of hemoglobin in bone and, consequently, the reaction’s intensity. For instance, unusually dry and hot environments may preserve hemoglobin longer, resulting in unexpectedly high intensities even in older remains. Therefore, low luminol intensity generally suggests an older PMI but cannot be interpreted as definitive without considering environmental context. These nuances reinforce the role of luminol as a presumptive test rather than a standalone determinant of postmortem interval. In applied forensic contexts, a positive luminol reaction may justify further analysis, while negative or borderline results should prompt careful consideration of burial and postmortem factors. The integration of contextual information remains essential for accurate interpretation. However, according to our results, the influence of such variability appears to diminish over time, with older samples converging towards consistently low Ipeak values, below 300 A.U.

Despite the variability observed at lower PMIs, the overall trend identified in the present study remains consistent. Samples with PMI below 20 years consistently yielded higher Ipeak values, while those with PMI exceeding 30 years remained below 300 A.U. (Table 2). These findings are in line with our previous research using visual classification systems [26], which reported that “strong positive” luminol reactions were only seen in remains up to 20 years PMI, while any reaction in remains beyond 30 years is mainly “Barely visible” or “Negative”. Our quantitative results not only reinforce those patterns but lend them greater accuracy by replacing categorical visual scoring with continuous numerical measurements. Compared to our previous studies [11, 21, 26], in which luminol chemiluminescence intensity was evaluated using both a binary and a five-level visual scale, the quantitative approach adopted in the present work allows for a more objective and reproducible assessment of the luminol technique. While the prior studies demonstrated the potential of luminol as a tool for discriminating between forensic and non-forensic remains, the method was inherently limited by its reliance on visual evaluation, although it yielded excellent inter and intraobserver agreement. The present findings not only confirm the trends observed in those earlier works but also enable a more refined statistical treatment of the data.

Our findings also align with previous research that employed a quantitative approach to estimate PMI in skeletonized remains through the luminol technique. Sarabia et al. (2018), using a luminometer to quantify luminescence in femora with PMIs ranging from 15 to 64 years, observed a significant inverse correlation between chemiluminescent intensity and PMI, particularly evident in the initial 10–20 s of the reaction. Moreover, their classification model achieved 88.4% accuracy in identifying remains older than 20 years, corresponding to the forensic relevance threshold applied in Spain, where the study was conducted. In contrast, the marked decrease in chemiluminescence intensity observed in the present study occurred beyond the ~ 30-year threshold identified through segmented modelling. Overall, the referred study confirmed the possibility of distinguishing human skeletonized remains of forensic interest through this quantitative and objective instrumental technique, reinforcing this approach as a more precise complement to the qualitative analysis previously studied, which is supported in the present investigation.

Nonetheless, as mentioned before, the luminol chemiluminescence technique should be interpreted as a presumptive screening tool rather than a standalone method for estimating PMI. The observed false positives (older remains exhibiting residual chemiluminescence) and potential false negatives (observed in previous studies) demonstrate the limitations of relying solely on luminol intensity. Accordingly, integrating contextual information is essential for accurate interpretation. In forensic casework, this technique can be particularly useful for the initial triage of human skeletonized remains, since a strong luminol reaction may prompt further forensic investigation, while a weak or absent one may suggest archaeological interest. Yet, a combination of methods, such as other screening techniques or confirmatory analysis, like radiocarbon dating, should be employed to strengthen the estimation of time since death.

Despite its potential, there are some limitations to this study, and to the luminol technique in general, that warrant discussion. Firstly, the small sample size, which consists of 24 individuals (23 used for statistical purposes), represents an inherent limitation of this study. As previously mentioned, this study was conceived as a pilot investigation introducing a new quantitative approach to measure luminol reaction intensity. For this reason, a smaller sample was intentionally used to explore methodological feasibility before expanding to larger datasets. Future studies on this matter should involve a larger sample to strengthen the robustness of the results. Nevertheless, despite the reduced sample size, PMIs are well distributed across the dataset, albeit with a notable temporal gap between 53 and 500 years postmortem. Secondly, as previously referred, and as in real forensic cases scenarios, the absence of detailed taphonomic contexts introduces uncontrolled variability, as factors such as burial environment, temperature and humidity exposure, and some individual characteristics were largely unknown. Thirdly, the analysis was restricted to clavicles, which may not fully capture inter-bone variability in the technique’s responsiveness. Finally, as a presumptive test, luminol is sensitive to iron-containing compounds but not entirely specific to human hemoglobin, leaving room for potential interference by other substances. These constraints highlight the need for cautious interpretation and underscore the importance of complementary data in forensic applications.

Overall, these results reinforce the utility of the luminol chemiluminescence technique as a rapid, cost-effective, and minimally destructive screening method for dating death, requiring only a small quantity of bone powder and producing results within minutes. The identification of a robust low-variation second segment and a statistically validated changepoint at approximately 30 years represents a significant step towards establishing luminol chemiluminescence as a presumptive screening tool for broad PMI estimation, determining whether remains are likely to fall within a forensic timeframe or are of archaeological interest. While accurate PMI estimation remains challenging, the results provide a conceptual and quantitative framework for building more refined models. However, the influence of taphonomic factors should be considered when interpreting individual results. Future research should aim to further investigate the influence of intrinsic and extrinsic factors and expand the dataset to include additional bone types and broader PMIs, improving the accuracy and applicability of this technique. Comparative validation with other emerging PMI estimation techniques should also be considered in future studies. With further refinement, the luminol technique can evolve from a promising presumptive test toward a more reliable, standardized technique in forensic anthropology.

## Conclusion

In conclusion, the present study supports the validity of quantifying the luminol chemiluminescence reaction for estimating PMI. Although limited in sample size, this pilot investigation provides essential preliminary evidence demonstrating the instrumental measurement of luminol-induced light emission as a feasible and promising approach for postmortem interval estimation as a presumptive screening tool. By quantitatively assessing the light intensity produced when powdered clavicle samples react with luminol, a clear inverse relationship between PMI and the Ipeak values was observed. The application of a segmented regression approach revealed a statistically supported changepoint at approximately 30 years postmortem, differentiating a highly variable early postmortem phase from a long-term phase characterized by consistently low and minimally variable Ipeak values. This threshold represents a key outcome of the present study and provides a practical framework for distinguishing remains of forensic interest from those of archaeological relevance.

The results from this quantitative approach mirror and reinforce the trends observed through previous qualitative visual scoring methods, while offering enhanced objectivity and statistical interpretability. These findings demonstrate that quantitative analysis provides advantages, reducing observer dependency and enabling more rigorous modelling of postmortem degradation patterns.

Although variability in early PMIs highlights the influence of taphonomic factors, the overall biphasic pattern supports the reliability of luminol chemiluminescence as a screening tool for PMI estimation. Future studies incorporating unstudied controlled decomposition contexts, larger sample sizes, and additional skeletal elements will be essential to further enhance the validity of the luminol chemiluminescence technique as an indicator of a more precise PMI estimation in human skeletonized remains. By contributing to the assessment of whether discovered skeletonized remains warrant forensic investigation, this technique may help streamline the investigative process and ensure that resources are appropriately allocated in the pursuit of the truth regarding the deceased. Ultimately, integrating quantitative luminol analysis with other forensic methods may contribute to a more robust, multidisciplinary framework for postmortem interval estimation, aligning scientific rigor with practical demands in forensic contexts.

## Data Availability

All data generated or analysed during this study are included in this published article.
